# Nutrition care for chronic kidney disease during pregnancy: an updated review

**DOI:** 10.1038/s41430-019-0550-6

**Published:** 2020-01-10

**Authors:** María Angélica Reyes-López, Giorgina B Piccoli, Filomena Leone, Alejandra Orozco-Guillén, Otilia Perichart-Perera

**Affiliations:** 1 Department of Nutrition and Bioprogramming, Instituto Nacional de Perinatología, Mexico City, Mexico; 2 Department of Clinical and Biological Sciencies, The University of Torino, Turin, Italy; 3 Hospital Cittá della Salute e della Scienza, Turin, Italy; 4 Department of Critical Medicine, Instituto Nacional de Perinatología, Mexico City, Mexico

**Keywords:** Nutrition, Kidney diseases, Kidney diseases, Nutrition, Nutrition

## Abstract

Cases of chronic kidney disease (CKD), including CKD in pregnant women, have increased globally in recent years. CKD during pregnancy is associated with a higher risk of adverse outcomes, including gestational hypertension, preeclampsia, intrauterine growth restriction, and preterm birth, among others. Nutrition plays a significant role in many metabolic and physiological changes during pregnancy. Women with CKD are at increased risk of nutrition deficiencies and metabolic issues than women without CKD. Currently, we lack evidence regarding metabolic and nutritional adaptations during pregnancy in women with CKD and how these adaptations relate to perinatal outcomes. In this review, dietary and supplementation recommendations for CKD in adults and pregnant women are summarized from current clinical guidelines. We present the main nutrition care practices that have been studied in CKD pregnancies. This review will be helpful to health professionals as a preliminary reference for nutrition assessment and therapy in pregnant women with CKD.

## Introduction

The frequency of chronic kidney disease (CKD) during pregnancy has increased. This could be related to the higher prevalence in CKD reported in recent years, the improvement of medical treatment in CKD adults, and a better kidney function assessment during pregnancy [1–3].

Pregnant women with CKD have specific nutritional needs and are at higher risk of adverse perinatal outcomes. There are currently no established guidelines to address nutrition therapy in this group. In this review, we aim to summarize established nutritional recommendations for nonpregnant individuals with CKD [4–6] and those for healthy pregnant women [7–9], and present data from studies done in pregnant women with CKD.

## Nutrition intervention for pregnant women with CKD

Nutrition therapy goals for pregnant women with CKD include to meet energy and nutrient needs for optimal fetal growth and development while promoting adequate metabolic control; to make individual recommendations for vitamin and mineral supplementation; and to promote a healthy lifestyle to improve maternal and fetal health.

### Energy and macronutrient recommendations in pregnant women with CKD

#### Energy recommendations

##### CKD

Resting energy expenditure (REE) appears to be higher in adults with CKD due to renal replacement therapy, poorly controlled diabetes mellitus (DM), severe hyperparathyroidism (HPT), and inflammation. In nondialyzed patients, REE tends to be equal or even lower than that of healthy individuals [10].

In stages 3–4 of CKD, the recommended energy intake is 96.2–146.4 kJ/kg/day (23–35 kcal/kg/day) using current weight [5]. During dialysis, American guidelines suggest to use 125.5–167.4 kJ/kg/day (30–35 kcal/kg/day), while European guidelines recommend 125.5–167.4 kJ/per ideal body weight/day (30–40 kcal/kg/day) [5, 6].

##### CKD and pregnancy

To estimate energy requirements in pregnant women, energy prediction equations can be used with pregestational weight, adding pregnancy energy needs (355.6 kJ/day or 85 kcal/day, 1192.4 kJ/day or 285 kcal/day, and 1987.4 kJ/day or 475 kcal/day during the 1st, 2nd, and 3rd trimester, respectively) [8, 7].

Gestational weight gain is an important indicator of energy balance and should be monitored. In healthy pregnancy, weight gain is determined by the pregestational body mass index. Institute of Medicine’s gestational weight gain recommendations range from 5 kg (in obesity) to 18 kg (in underweight). The Intergrowth Consortium suggests a gestational weight gain of 13.6 kg in normal-weight women [11, 12].

In women with advanced CKD stages, weight gain assessment is a challenge. Data from dialysis patients have reported a weight gain of 1–1.5 kg during the first trimester and 0.5 kg/week in the second and third trimesters [13]. Several reports of pregnant women undergoing dialysis with optimal perinatal outcomes showed a total weight gain between 5.2 and 12 kg, with the highest gain in patients who received nutrition therapy [14–16].

In CKD pregnant women in stages 1 and 2, it seems prudent to use energy intake recommendations for healthy pregnant women. During stages 3 and 4, energy recommendations for nonpregnant CKD adults could be used, using pregestational weight and adding the energy cost of pregnancy. If pregestational weight is not reliable, current dry weight can be used without adding pregnancy requirements. For pregnant women under HD and peritoneal dialysis (PD), the Italian study group suggests 146.4 kJ/kg (35 kcal/kg) and 104.6 kJ/kg (25 kcal/kg) with pregestational weight, respectively, and adding the energy cost of pregnancy [17]. Reported cases of pregnancies undergoing HD showed that an intake of 125.5–146.4 kJ/pregestational (30–35 kcal/kg) plus 1255.2–1674.4 kJ (300–400 kcal/day) from the second trimester was positively associated with maternal weight gain, lower incidence of small-for-gestational age (SGA), and less frequent neonatal intensive care unit hospitalization [14–16, 18, 19].

#### Protein recommendations

##### CKD

For adults with CKD with a glomerular filtration rate (GFR) below 50 ml/min per 1.73 m^2^, the protein intake recommendation is 0.6–0.8 g/kg/day. When GFR is <20 ml/min per 1.73 m^2^, a very low-protein diet may be prescribed (0.3–0.5 g/kg/day) with ketoacid analog supplementation [5].

In adults under dialysis, protein intake recommendations range from 1.1 to 1.3 g/kg/day, using ideal or current dry body weight. Protein intake should be increased with more intensive HD [6]. At least 50% of dietary protein should be of high biological value [4, 6].

Signs, symptoms, and markers related to uremic syndrome and renal function (GFR, BUN, serum creatinine, and proteinuria) should be monitored to individualize protein intake recommendations.

##### CKD and pregnancy

Protein requirements in pregnancy are 71 g/day or 1.1 g/kg/day. Adult protein requirements can be used (0.8 g/kg of pregestational weight/day) if additional protein needs of pregnancy are added (+1, 9, and 31 g/day during the 1st, 2nd, and 3rd trimester, respectively) [7, 9].

For pregnant women in CKD stages 1 and 2, recommended protein intake may be similar to that for a healthy pregnancy. In advanced CKD stages and under dialysis, protein recommendations for adults with CKD may be used, while adding the extra needs of pregnancy. In stages 3–5, a moderate-protein-restricted plant-based diet (0.6–0.8 g/kg/day of protein) plus keto-analogs (1 tablet/8–10 kg) showed a lower frequency of early preterm delivery, very low birth weight (LBW) (<1500 g) and SGA infants [20–23]. In different reported case series of pregnant women under HD, a protein intake of 1.2–1.5 g/kg of ideal body weight/day plus 6–10 g/day was related with optimal plasmatic nitrogen compounds levels, controlled blood pressure, reduced proteinuria, and improved plasmatic albumin and phosphorus [14, 15, 18]. Vidal et al. observed an optimal maternal weight gain, good calcium–phosphate metabolism, and favorable neonatal outcomes with 1.1–1.2 g/kg/day (pregestational dry weight/day) of protein during the first trimester and 1.5 g/kg (current body weight) in the second and third trimesters [16]. Espinoza et al. recommended a higher amount of protein (1.8–2 g/kg/day) and added intradialytic amino acids in cases of low intake, observing a lower incidence of SGA infants [19]. According to the Italian study group, protein intake recommendations for HD and PD should be 1.2 and 1.4 g/kg/day pregestational weight, respectively [17].

#### Lipid recommendations

##### CKD

CKD is associated with dyslipidemia, showing similar characteristics in early and advanced kidney failure [low high-density lipoproteins, normal or low total cholesterol, high low-density lipoprotein cholesterol and increased triglycerides (TG)]. Lipid profile in patients with PD tends to be more atherogenic [24, 25].

Lipid and saturated fat intakes for individuals with CKD should be maintained below 30 and 10% of total energy intake (TEI), respectively [25]. In hypertriglyceridemia, a low-fat diet (<15% TEI) is suggested with an accompanying reduction in mono and disaccharide intake and the use of fish oil to replace long-chain TGs [25]. In case of hypercholesterolemia, monounsaturated fat recommended intake is >10%, saturated fatty acid <10%, and cholesterol intake <300 mg/day [6].

##### CKD and pregnancy

Lipid metabolism during pregnancy is characterized by an accumulation of fat in maternal depots during early pregnancy and the later development of hyperlipidemia due to increasing lipolysis and mobilization of TG [26].

No lipid status has been reported in CKD pregnant women. In our case series of pregnant women with CKD (without dialysis), mean serum cholesterol and TG were 246.6 ± 116.7 mg/dL and 277.0 ± 187.0 mg/dL, respectively, in the second trimester, and 277.1 ± 129.3 mg/dL and 341.1 ± 190.6 mg/dL, respectively, in the third trimester (*n* = 48) (unpublished data).

The total fat intake recommendation in pregnancy is <30–35% of TEI [7]. Omega 3 fatty acids play an important role in retinal and neurological fetal development. European recommendations for EPA + DHA are 250 mg/day and an extra 100–200 mg/day of DHA [27]. Linoleic and α-linolenic fatty acids intake recommendations are 13 g/day and 1.4 g/day, respectively [7, 27, 28]. Oily fish (salmon, trout, sardines, and tuna) consumption should be increased during pregnancy (250–300 g/week) [7, 8].

#### Carbohydrate recommendations

Glucose metabolism is frequently impaired in CKD due to reduced insulin-mediated glucose uptake in skeletal muscle. Insulin resistance is more frequent in advanced CKD stages. Although some patients can compensate for insulin resistance by increasing insulin secretion, defects in insulin secretion are common [29].

In adults with CKD and DM, carbohydrate intake recommendations should be addressed to manage hyperglycemia, aiming to achieve glycemic control (HbA1C < 7%) [5].

Early pregnancy is characterized by an increase in insulin sensitivity, while an insulin resistance state develops during late pregnancy to prioritize the use of fats for energy, sparing carbohydrates for the fetus [30]. If CKD pregnant women have previous DM or GDM, glycemic control is the main goal of treatment (goal:fasting capillary glucose ≤ 95 mg/dL and 1-h postprandial capillary glucose ≤140 mg/dL) [31].

In a healthy pregnancy, acceptable carbohydrate recommended intake is 45–65% of TEI [28], while fiber intake recommendation is 28 g/day [28]. In women with previous DM or GDM, carbohydrate recommendations should be individualized [31]. The most accepted strategies include monitoring carbohydrate intake (‘carb counting’). Three meals and 2–3 snacks are preferred while promoting healthy carbohydrate consumption (low-glycemic index foods and/or high-fiber food sources) and avoiding added sugars and processed foods [32, 33]. A minimal carbohydrate intake of 175 g/day during pregnancy seems appropriate [28].

#### Fluids

CKD is associated with hydroelectrolytic disturbances. When GFR is below 20 mL/min, the kidney loses its capacity to concentrate or dilute the urine [34]. Sodium and water recommendations should be individualized based on diuresis and interdialytic weight gain in dialysis patients. Interdialytic weight gain has been suggested to be 4–4.5% of dry weight [5]. Fluid intake recommendation for a healthy pregnancy is 3 L/day [28]. Table 1 summarizes energy and macronutrient recommendations for pregnant women with CKD.

**Table 1:** Macronutrient recommendations for CKD pregnant women.

	Trimester	CKD stages	
		CKD 3–5 stages	Dialysis
Energy (kJ/kg/day)^a^	First	96–146 + 289 kJ	105–146 + 289 kJ^b^
	Second	96–146 + 1100–1423 kJ	105–146 + 1100–1423 kJ^b^
	Third	96–146 + 1891–2096 kJ	105–146 + 1891–2096 kJ^b^
Protein (g/kg/day)^a^	First	0.6–1.0 + 0.7 g	1.1–1.5 + 0.7 g
	Second	0.6–1.0 + 9.6 g	1.1–1.5 + 9.6 g
	Third	0.6–1.0 + 31.2 g	1.1–1.5 + 31.2 g
Carbohydrates % TEI		35–65	
Fiber (g/day)		20–30	
Lipids % TEI		≤30	
Saturated fatty acids % TEI		<7–10^c^	
Monounsaturated fatty acids % TEI		10–20^d^	
Polyunsaturated fat % TEI		≤10	
Linoleic acid (g/day)		13	
α-Linolenic acid (g/day)		1.4	
DHA (mg/day)		200	
Cholesterol (mg/day)		<200–300	

^a^Pregestational body weight

^b^Due glucose absorption from dialysate, the lower range is suggested for peritoneal dialysis

^c^In case of dialysis apply 7% of TEI

^d^In case of dialysis apply 20% of TEI. From references [4–9, 27, 28]

#### Vitamin and mineral recommendations

In early CKD stages, micronutrient intake recommendations are similar to those for a healthy pregnancy. Specific recommendations are now needed for those at more advanced stages of CKD and pregnant women on dialysis. Table 2 summarizes vitamin and mineral recommendations for healthy pregnant women and for nonpregnant CKD individuals.

**Table 2:** DRI and DRV of micronutrients for healthy pregnant women over 18 years and for nonpregnant CKD individuals.

Nutrient	Pregnancy	Nonpregnant CKD individuals	
	Healthy pregnancy (DRI-DRV)^a^	Pre-ESRD	Dialysis
Calcium (mg)	950–1000	<2000	<1500–2000
Phosphorus (mg)	550–700	800–1000	800–1000
Potassium (mg)	3500–4700	≤2400	3700–5200
Sodium (mg)	1500	<2400	2000–2300
Iron (mg)	16–27	15^c^	15^c^
Zinc (mg)	11–12^d^	8–12	8–12
Magnesium (mg)	300–350	290–300	200–300
Vitamin D (UI)	600	800–1000	800–1000^f^
Vitamin A (µg)	700–770	550	700–900
Vitamin C (mg)	85–105	30–60	75–90
Vitamin B1 (mg)	0.8–1.4^g^	1.1	1.1–1.2
Vitamin B2 (mg)	1.4–1.9	1.1	1.1–1.3
Vitamin B3 (mg)	13.4–18^g^	14	14–16
Vitamin B6 (mg)	1.8–1.9	5	10
Vitamin B12 (µg)	2.2–4.5	>2–3	2.4
Biotin (µg)	30–40	30–100	30
Folic acid (mg)	0.6	>1	1.0

*DRI* dietary references intakes, *DRV* dietary references values, *ESRD* end-stage-renal disease

^a^Recommended dietary allowance (RDA) and Populations reference intake (PRI)

^b^Pregestational body weight

^c^According iron studies and erythropoiesis-stimulating agent use

^d^Based on the average for zinc recommendation according phytates consumption and the amount add for pregnancy

^e^1,25(OH)2D3 if low serum levels

^f^Adjust dose based on phosphorus, calcium, PTH levels

^g^For each 2000 kcal. From refs. [4, 5, 27, 28]

#### Calcium, vitamin D, and phosphorus

##### CKD

CKD is characterized by disturbances in mineral, vitamin D, and parathyroid hormone (PTH) metabolism [35]. As GFR declines, a fall in the activity of the renal vitamin D 1α-hydroxylase enzyme is observed. As a result, the conversion of vitamin D to its active form, 25-hydroxyvitamin D (25-OH-D), is impaired. Consequently, vitamin D-dependent calcium absorption is limited, hypocalcemia occurs, and PTH production increases in a counterregulatory attempt to release calcium from bone stores. In contrast, phosphorus excretion is decreased, triggering an increased production of fibroblast growth factor, which also suppresses 1α-hydroxylase. The release of bone mineral stores exacerbates hyperphosphatemia, which promotes further production of PTH [35, 36].

KDIGO recommends measuring serum 25-OH-D, calcium, phosphorus, PTH, and alkaline phosphatase activity at least once in adults with CKD stage 3 and above, and then annual reassessment if levels are within normal parameters [3]. Clinical signs and symptoms of hyperphosphatemia (Itching, bone pain, and vascular calcification) related to the advanced stages of CKD should be assessed and monitored [36].

During stages 3–5 and HD, the Academy of Nutrition and Dietetics suggests a total elemental calcium intake (from the diet, supplements, and phosphate binders) <2 g/day in CKD patients [5].

Dietary phosphorus should be restricted to 800–1000 mg/day when serum phosphorus levels are >4.6 mg/dL (stages 3–4) or >5.5 mg/dL (stage 5) [5]. Dietary phosphorus restriction is also needed when plasma PTH is >70 pg/ml in more than two consecutive measurements. Dietary strategies for phosphorus restriction include to avoid organic (i.e., dairy, meats, poultry, fish, and grains) and inorganic (preservatives and additives) sources [36].

Vitamin D supplementation with 800–1000 IU of cholecalciferol is indicated when serum 25-OH-D is below 30 ng/ml or in stages 4–5 with severe and progressive HPT [36].

In stages 3–4, active oral supplementation with calcitriol or vitamin D analogs is indicated when plasma PTH is high and in patients with serum corrected total calcium <9.5 mg/dL and serum phosphorus <4.6 mg/dL [5].

##### CKD and pregnancy

During a healthy pregnancy, calcium absorption is stimulated to meet increased requirements [37]. Increased production of 1,25[OH]_2_D secondary to 1-alpha-hydroxylase placental activity is observed. Vitamin D homeostasis is characterized by an increase in maternal calcitriol, enhanced availability of maternal 25-OH-D, and increase of maternal vitamin D-binding protein concentrations [37]. The American College of Obstetricians and Gynecologists considers levels of circulating 25-OH-D below 32 ng/mL during pregnancy as deficient [38].

There are no reports of serum PTH and 25-OH-D concentrations in CKD pregnant women. In our case series of pregnant women with CKD (stages 1–4 without dialysis), we observed PTH levels in the second and third trimesters of 55.3 ± 107.6 pg/ml and 47.7 ± 36.9 pg/ml, respectively. These concentrations exceed normal ranges for a healthy pregnancy but are within normal ranges reported in CKD adults (Table 3). Plasma 25-OH-D concentrations were 27.6 ± 11.7 ng/ml (second trimester) and 28.5 ± 10.7 ng/ml (third trimester). During the second and third trimesters, 22% (*n* = 8/36) and 19% (*n* = 5/26) of women had low 25-OH-D (<20 ng/mL), respectively. We observed a positive correlation between PTH net change from the second to the third trimester with plasma phosphorus concentration in the third trimester (*r* = 0.43, *p* = 0.020) (unpublished data). Table 3 shows the reference values for bone mineral metabolism biochemical markers in CKD and during healthy pregnancy.

**Table 3:** Reference values for biochemical markers assessment in CKD and in pregnancy.

	Women with CKD	Pregnancy without CKD		
		First trimester	Second trimester	Third trimester
**Bone mineral metabolism**				
Calcium (mg/dL)	9–10.5	8.8–10.6	8.2–9.0	8.2–9.7
Phosphorus (mg/dL)	3–4.5	3.1–4.6	2.5–4.6	2.8–4.6
Vitamin D 25 hydroxycholecalciferol (pg/mL)	30–65	20–65	72–160	60–119
Parathyroid hormone (pg/mL)	Stage 3: 35–70 Stage 4: 70–110 Stage 5 and dialysis: 150–300	10–15	18–25	9–26
Alkaline phosphatase (IU/L)	30–85	17–88	25–126	38–229
**Iron status**				
Hemoglobin (g/dL)	11–12	≥11.0	≥10.5	≥11.0
Hematocrit (%)	37–47	≥33	≥32	≥33
Serum iron (µg/dL)	50–170	72–143	44–178	30–193
Ferritin (ng/mL)	Not dialysis >30 HD ≥ 20—PD > 100	6–130	2–230	0–116
Total iron binding capacity (TICB) (µg/dL)	250–420	278–403	–	359–609
Transferrin saturation (%)^a^	≥20	Not reported	18–92	9–98

*HD* hemodialysis, *PD* peritoneal dialysis

^a^Measure when clinically stable, not measure in acute illness. Pregnancy references adapted from: Abbassi-Ghanavati, Greer, Cunningham. Pregnancy and Laboratory Studies. Obstet Gynecol. 2009;114:(6)1326–31. From refs. [36, 42]

In healthy pregnant women with adequate vitamin D stores and normal PTH levels, calcium intake should be 1000–1300 mg/day [27, 39]. The WHO recommends 1.5–2.0 g/day of elemental calcium in women with a high risk of developing preeclampsia (i.e., CKD) and with low-calcium intakes [40]. Phosphorus intake recommendation for healthy pregnancy is 700–1250 mg/day [27, 39]. High prevalence of low serum 25-OH-D are frequently reported during pregnancy, so 25-OH-D assessment seems important to decide if supplementation is necessary [37]. Vitamin D doses of 1000–2000 IU/day as supplements could be considered safe during pregnancy [38].

Calcium supplementation has been reported in isolated case studies, ranging from 300 to 1500 mg/day [14, 16].

Due to more frequent dialysis sessions, phosphorus restriction, and phosphate binders use might not be needed in CKD pregnancies [2]. Although oral vitamin D supplementation is suggested in CKD pregnant women, very low doses have been used (10–250 UI/day) [14, 16]. Vitamin D analogs have been given intravenously to pregnant women undergoing dialysis, but it is not clear whether they have any adverse effect on the fetus. Once 25-OH-D reaches its optimal levels, a vitamin D-containing multivitamin should be continued [41].

#### Iron, folic acid, and cobalamin

##### CKD

Anemia in women with CKD is diagnosed when hemoglobin levels are below 12.0 g/dL, and it is associated with insufficient production and release of erythropoietin (EPO), so in most cases, anemia is characterized as being hyperproliferative, normochromic, and normocytic [42]. Besides insufficient EPO production, iron deficiency is the most common reversible cause of chronic anemia in CKD patients. An in-depth assessment that includes a complete blood count, absolute reticulocyte count, serum ferritin level, serum transferrin saturation (TSAT), serum vitamin B12, and folate levels should be performed [42]. KDIGO suggests monitoring nonanemic CKD patients when clinically indicated or annually (stage 3), twice per year (stages 4–5 without dialysis), or every 3 months (stage 5 with dialysis). Iron status should be monitored every 3 months with more frequent assessments in individuals on HD and receiving erythropoiesis-stimulating agents (ESA) [43].

KDIGO recommends IV or oral iron trials in adults with or without ESA therapy if the goal is to increase Hb without starting ESA treatment, or to decrease ESA dose, and if transferrin saturation is <30% and ferritin are <500 ng/ml [42]. For HD individuals, the recommendation is 50–60 mg of intravenous iron per week [43]. Cobalamin and folic acid supplementation are recommended if the mean corpuscular volume is >100 ng/ml and if serum levels of these nutrients are below normal values [5].

##### CKD and pregnancy

The World Health Organization (WHO) defines anemia during pregnancy as Hb < 11 g/dL or hematocrit < 33% at any time during the pregnancy [44]. Physiologic anemia of pregnancy reflects an expansion of plasma volume of 50%. Globally, the most common cause for anemia of pregnancy is iron deficiency and is related to increased iron requirements. Iron-deficiency anemia is associated with higher rates of preterm birth, LBW, and SGA newborns [44].

Inadequate intake of folic acid is associated with a higher risk of neural tube defects, LBW, and SGA. Maternal vitamin B12 status affects fetal growth and development, and its low levels are associated with some metabolic alterations and impaired neurodevelopment [44]. In CKD pregnant women, anemia might also be related to a higher risk of prematurity, LBW, and adverse perinatal outcomes [1]. Table 3 shows the reference values for iron status markers in CKD and during a healthy pregnancy.

The WHO recommends supplementing 30–60 mg/day of elemental iron daily and 400 µg/day of folic acid starting 3 months before pregnancy. In the case of anemia, 120 mg/day of oral iron is recommended [45, 46]. Iron IV supplementation is considered when oral iron intolerance is reported in severe anemia (<8 g/dL) [44]. Cobalamin supplementation is not routinely recommended except for vegetarian/vegan women or those with a very low intake of animal products [8].

Oral iron supplementation is safe in CKD pregnancies under HD. IV iron might be used in dialysis during pregnancy with caution. Cabiddu suggests to use 20–30 mg/day of IV elemental iron (iron gluconate) until transferrin saturation reaches 30% and serum ferritin 200–300 ng/mL [17]. Folic acid needs are higher in CKD pregnancies; supplementation has been recommended with doses ranging between 1 and 5 mg/day, considering the higher doses for pregnant women receiving HD [2, 41].

#### Sodium and potassium

In CKD adults, sodium and potassium recommendations should be individualized based on serum concentrations, blood pressure, medications, hydration status, and kidney function. Clinical signs and symptoms of hyperkalemia (tremor, muscle weakness, dyspnea, bradycardia, arrhythmia, and nausea) should be assessed and monitored. In hyperkalemic adults with CKD, dietary intake of potassium <2.4 g/day is recommended. Restriction of some fruits, vegetables, and other foods rich in potassium (processed foods with chemical potassium compounds) might be needed [5].

In CKD, sodium intake recommendation should be maintained at <2.4 g/day by limiting the consumption of processed meats, smoked foods, ready-to-use seasonings and dressings, instant soups, preservatives, and salt additives [5].

In a group of pregnant women under HD (18–24 h/week), Vidal et al. offered 3–4 g/day of salt and 3 g of potassium, among other recommendations, reporting positive perinatal outcomes [16].

## Conclusions

Given the lack of specific research on this population, it is possible to make adaptations to the guidelines used for healthy pregnant and for nonpregnant individuals with CKD. The role of the renal nutritionist is key in tailoring energy and nutrient intakes to promote maternal and fetal health and metabolic control. More research is urgently needed to develop nutritional guidelines for this population.
